# Blood-panel patterns across feline creatinine strata and shared baseline effects on ratio associations

**DOI:** 10.3389/fvets.2026.1972280

**Published:** 2026-10-22

**Authors:** Hocheol Shin, Jae Young Kim, Jung Eun Yun, Seung Rok Kang, Junha Jang, Motoo Kobayashi

**Affiliations:** 1 Greycoat Research Inc., Seongnam-si, Republic of Korea; 2 Taeneung Animal Hospital, Seoul, Republic of Korea; 3 Seijo Kobayashi Veterinary Clinic, Setagaya, Tokyo, Japan

**Keywords:** creatinine, creatinine strata, feline chronic kidney disease, inflammaging, phosphorus-to-creatinine ratio, routine blood panel, serum amyloid A

## Abstract

**Introduction:**

Cats with similar creatinine (CRE) values can have different accompanying blood-panel findings, but the relevance of these patterns to subsequent change is uncertain.

**Methods:**

We examined routine blood panels across seven clinically proposed creatinine strata, refined through retrospective review. The dataset comprised 5,743 episode-anchored visits from 3,006 cats; chronic kidney disease (CKD) diagnoses were unavailable. A broad candidate review yielded 33 calculable markers for screening.

**Results:**

The ratios of blood urea nitrogen (BUN), phosphorus (PHOS), and symmetric dimethylarginine (SDMA) to CRE met false-discovery-rate correction for association with creatinine slopes that included the baseline visit. None met correction for slopes using subsequent visits only. Removing the baseline visit reduced absolute correlations more for these three ratios than for 27 other nonredundant markers (mean difference 0.120; cat-cluster bootstrap 95% CI 0.038–0.198), consistent with shared-baseline coupling. In *post hoc* analyses of strata 1–2, PHOS/CRE and SDMA/CRE associations remained positive after adjustment for age and baseline creatinine, but did not meet correction (both q = 0.072). Component models did not show consistent added value from either ratio. The BUN-to-albumin (ALB) ratio mainly reflected current values and within-episode changes. Serum amyloid A (SAA), the neutrophil (NEU)-to-lymphocyte (LYM) ratio, and the ALB-to-globulin (GLOB) ratio described systemic patterns weakly associated with creatinine.

**Discussion:**

Clinical observations and retrospective data supported considering accompanying systemic and electrolyte findings when assessing individual cats. Whether interpreting these findings together improves clinical assessment requires prospective validation.

## 1 Introduction

Chronic kidney disease (CKD) is one of the major causes of morbidity and mortality in older cats, and its prevalence increases markedly with age (1). Feline CKD is characterized by the progressive loss of functional nephrons, leading to impaired excretory, endocrine, and homeostatic functions. Because obvious clinical signs often become apparent only after a substantial reduction in renal reserve, early detection and clinically meaningful risk stratification remain important goals in feline CKD management. Several prognostic factors have been reported in affected cats, including the degree of azotemia, proteinuria, and other determinants of survival (2–4).

The International Renal Interest Society (IRIS) staging system is widely used for classifying CKD severity, after CKD has been diagnosed, based mainly on stable blood creatinine (CRE) concentration, with symmetric dimethylarginine (SDMA) used as an additional marker when appropriate (5, 6). Its strengths are that it is simple, reproducible, and already familiar to clinicians. Within a broad creatinine range, however, cats can differ in their accompanying laboratory findings and the issues that require attention during follow-up. Creatinine and SDMA primarily reflect filtration and do not directly describe all systemic changes that may accompany CKD. Creatinine can be affected by lean body mass and hydration status (7), whereas SDMA, although less dependent on muscle mass and correlated with glomerular filtration rate, remains a filtration-related marker (8–10). Other markers, such as cystatin C, have been studied in small animals but are not yet included in routine feline CKD staging (11).

The present study was based on two considerations. First, routine blood panels contain relationships among co-measured analytes that may be less apparent when each result is read separately. Concordance, or discordance, between kidney-related biomarkers may itself provide useful information (12). For example, phosphorus (PHOS) and creatinine reflect related but different aspects of renal function and mineral handling. PHOS/CRE asks how much phosphorus accompanies a given creatinine value. SDMA/CRE compares the relative levels of two filtration markers, while the blood urea nitrogen (BUN)-to-albumin (ALB) ratio places azotemia in the context of a protein measurement. These ratios are simple to calculate, but their interpretation depends on whether they provide information beyond their components. For CRE-denominator ratios, baseline CRE may also contribute to both the ratio and the slope, affecting their apparent association.

Second, the current condition of a cat may change without a parallel change in creatinine. Serum amyloid A (SAA), a major positive acute-phase protein in cats (13), the neutrophil (NEU)-to-lymphocyte (LYM) ratio, and the ALB-to-globulin (GLOB) ratio provide information about accompanying systemic findings. A previous study reported higher SAA in cats with IRIS stage 2–4 CKD than in healthy controls (14), consistent with interest in inflammaging (15) and age-related metabolic and inflammatory changes in cats (16). In the clinical experience that motivated this study, these findings were reviewed alongside renal measurements when assessing illness and changes over repeated visits. Electrolyte and mineral abnormalities also deserved attention because they could affect the interpretation of an individual cat’s condition. We included these measurements for their clinical relevance, even when they did not closely track creatinine.

Our starting point was the clinical observation that cats within the same broad creatinine range can show different accompanying blood-panel patterns. We translated these observations into seven proposed strata and reviewed them against retrospective data, using IRIS creatinine ranges as a clinical reference (Figure 1). Previous work has developed a routine-panel model for early feline CKD risk (17). Here, we asked how accompanying analytes varied across creatinine ranges and whether selected ratios related to subsequent creatinine change beyond their component measurements. We reviewed a broad set of candidate blood-panel markers and screened 33 that could be calculated directly from the available data. Selected markers were then examined in greater detail according to their clinical relevance and observed patterns.

**Figure 1:**
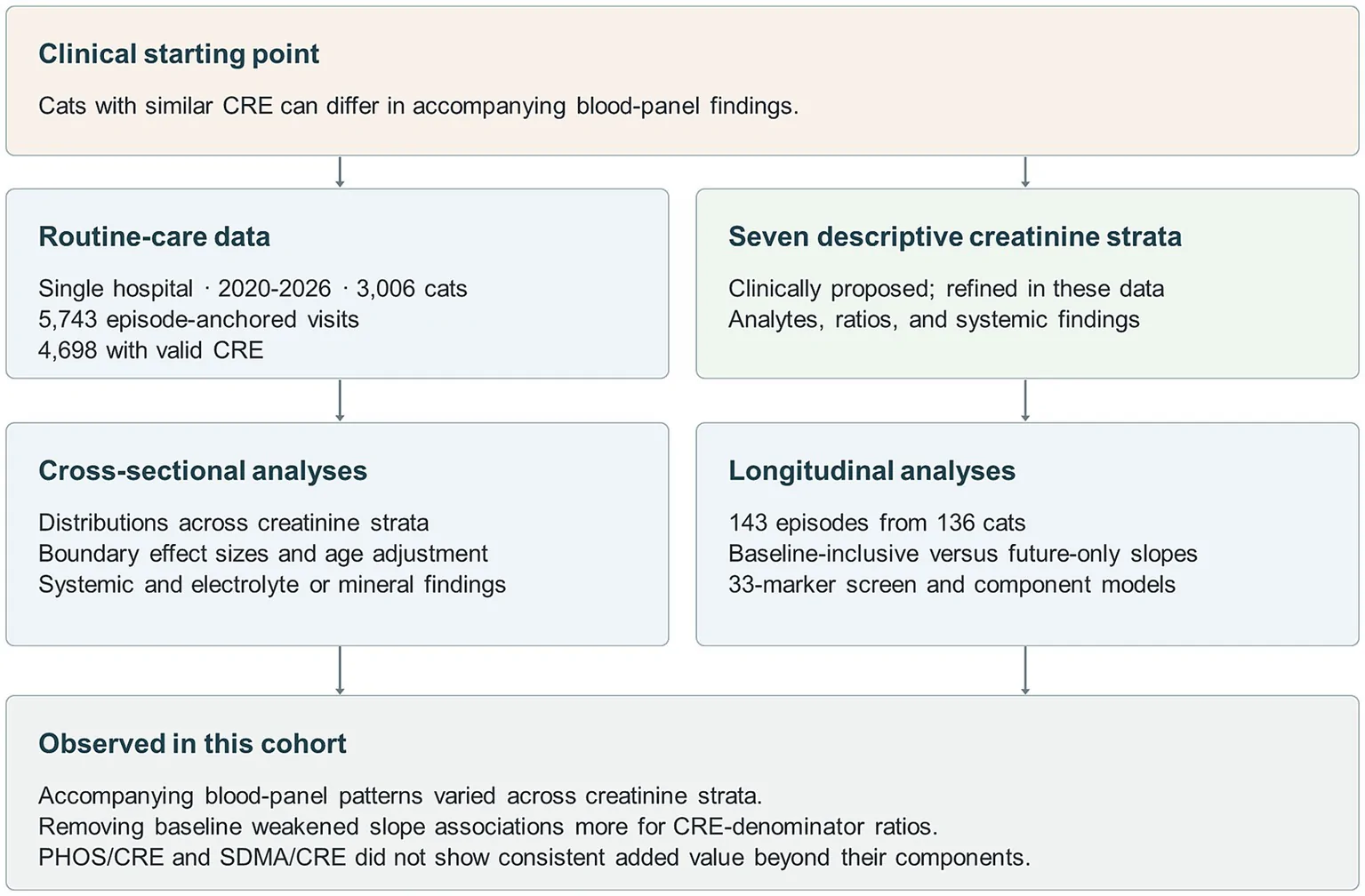
Clinical starting point and analytical overview. Clinical observations guided seven provisional creatinine strata and the choice of accompanying blood panel measurements. Cross-sectional summaries used the first three visits per episode; longitudinal slopes used all eligible visits. Comparison of slope definitions and component models assessed the interpretation of derived ratios. CRE, creatinine; BUN, blood urea nitrogen; PHOS, phosphorus; SDMA, symmetric dimethylarginine.

## 2 Materials and methods

### 2.1 Study population and data source

Blood-panel records were obtained from the electronic medical records of a single referral animal hospital from 2020 to 2026, comprising 7,094 visits with valid dates and patient identifiers. The hospital reported using Catalyst One with a Chemistry 15 CLIP panel (IDEXX Laboratories) for biochemistry, IDEXX ProCyte Dx for hematology, and Skyla Solution for SAA. SDMA was ordered separately; the historical assay platform and reference intervals could not be verified. Clinical diagnoses were unavailable, and the study population included cats receiving routine care rather than a cohort restricted to confirmed CKD cases. Values affected by suspected test-field errors were set to missing before derived markers were calculated. A urine specific gravity (USG) field was present in 92 of 93 suspect CRE entries, compared with 160 of 7,094 visits overall (2.26%), supporting suspected test-field mixing without confirming its origin. Data-cleaning procedures are described in Supplementary Methods 1.1.

### 2.2 Episode-anchored sampling

Repeated visits from the same cat can lead to pseudoreplication if treated as independent observations. To limit the contribution of frequently monitored cats, we defined a new care episode after a gap of more than 180 days and retained the first three visits per episode for cross-sectional analyses. This reduced 7,094 raw visits to 5,743 visits from 3,006 cats.

Longitudinal analyses used marker values at the first visit of episodes with at least four valid creatinine measurements over at least 0.5 years. We calculated a baseline-inclusive slope using all eligible visits and a future-only slope using subsequent visits, with at least three measurements on at least two dates. Comparing these slopes assessed the influence of including the same baseline measurement in both the marker and the outcome. Secondary analyses examined lower (strata 1–2) and higher (strata 3–7) creatinine ranges. Longitudinal slopes used all eligible visits, without the first-three-visit cap. Detailed sampling and slope procedures are given in Supplementary Methods 1.2; comparisons of follow-up and measurement availability are described in 1.3.

### 2.3 Creatinine strata and accompanying blood-panel measurements

Seven creatinine strata were used to describe accompanying blood-panel patterns, with approximate reference to the broader IRIS creatinine ranges (Table 1). Candidate boundaries came from clinical experience and were finalized after reviewing analyte patterns in this cohort. The boundaries are therefore provisional and were not validated against a clinical outcome. These strata describe creatinine ranges and do not establish CKD diagnoses.

**Table 1:** Proposed creatinine strata, approximate correspondence to IRIS creatinine-ranges, and distribution of visits.

Creatinine stratum	CRE range (mg/dL)	Approximate IRIS creatinine range	*n*	% of stageable visits
1	<1.7	Below/IRIS 1 range	2,474	52.7%
2	1.7–2.3	IRIS 2 range	1,607	34.2%
3	>2.3–2.9	IRIS 2 range	307	6.5%
4	>2.9–3.5	IRIS 3 range	91	1.9%
5	>3.5–5.0	IRIS 3 range	87	1.9%
6	>5.0–7.0	IRIS 4 range	40	0.9%
7	>7.0	IRIS 4 range	92	2.0%

Percentages were calculated among 4,698 visits with valid CRE. The proposed ranges do not have identical boundaries to IRIS ranges (for example, CRE 1.6 and 2.9 mg/dL) and do not establish an IRIS CKD diagnosis.

PHOS/CRE, SDMA/CRE, and BUN/ALB summarized relationships between routine analytes. SAA, NEU/LYM, ALB/GLOB, and hematocrit (HCT) described accompanying systemic and hematologic findings. Potassium (K) and the calcium (Ca)-phosphorus product (Ca × PHOS) were examined separately for electrolyte and mineral abnormalities. Hypokalemia was defined as potassium <3.5 mmol/L; Ca × PHOS >70 mg^2^/dL^2^ was used as an operational caution flag, not a validated intervention threshold. Stratum comparisons and figure summaries are detailed in Supplementary Methods 1.4.

### 2.4 Candidate marker discovery screen

We reviewed candidate ratios and other markers derived from routine blood panels and screened the 33 that could be directly calculated from the available measurements. Each marker was assessed for its cross-sectional association with creatinine, association with creatinine slope, and tracking of creatinine within episodes. Selected markers were examined further in relation to the clinical questions motivating this study and their observed patterns. The screen and subsequent analyses were exploratory. Marker definitions and screening results are provided in Supplementary Table S1; screening procedures and candidate selection are detailed in Supplementary Methods 1.5, 1.6.

To assess shared-baseline coupling, we compared the reduction in absolute slope correlations after removing the baseline visit for creatinine-denominator ratios versus other markers. This comparison used 30 nonredundant markers and a cat-cluster bootstrap confidence interval (Supplementary Methods 1.6).

### 2.5 Statistical analysis

Spearman correlations assessed monotonic associations, and ordinary least squares estimated creatinine slopes in mg/dL/year. Partial rank correlations adjusted for age and baseline creatinine. Inference accounted for within-cat clustering using cat-cluster-robust standard errors or bootstrap confidence intervals. Benjamini-Hochberg false-discovery-rate (BH-FDR) correction addressed multiple testing within the defined analysis families; *p* < 0.05 indicated nominal significance and q < 0.05 indicated significance after correction. Rank models, correction families, and age-adjusted boundary comparisons are detailed in Supplementary Methods 1.7. Systemic-marker comparisons used available paired measurements and were repeated with age adjustment (Supplementary Methods 1.3, 1.8).

We tested the contributions of baseline PHOS and SDMA to future-only slope models containing age and baseline CRE, then tested whether the corresponding ratio added information beyond these components. Rank-based models were compared with models using measurements on their original scales. Cross-validation kept cats separate between training and testing. We also compared seven-stratum, four-range, and continuous-creatinine models descriptively (Supplementary Methods 1.9). Sensitivity analyses used alternative sampling and cleaning rules, Theil-Sen and first-to-last slopes, and longer follow-up requirements (Supplementary Methods 1.1, 1.2, 1.10). We also examined baseline CRE-slope correlations and summarized measurement counts, follow-up duration, slope distributions, and individual regression fit. Reproducible analyses and aggregate outputs are provided in Supplementary Code.

Analyses used Python with pandas, SciPy, statsmodels, and scikit-learn. Figures were prepared in GraphPad Prism 8. OpenAI ChatGPT and Codex assisted with analytical code development and statistical cross-checking; all analytical decisions and outputs were reviewed by the authors.

### 2.6 Ethics statement

The animal study was reviewed and approved by the MoMo Group Research Animal Ethics Committee. The committee is part of MoMo Group Inc.; JYK is affiliated with both the company and Taeneung Animal Hospital, which provided the records. This study used retrospective, anonymized clinical records from client-owned cats that were generated during routine veterinary care. No additional procedures were performed for research purposes. The requirement for additional consent was waived by the ethics committee because the study was retrospective, used anonymized clinical records, and involved no additional animal procedures. The study was conducted in accordance with applicable local legislation and institutional requirements.

## 3 Results

### 3.1 Blood-panel differences across creatinine strata

Clinical experience and retrospective review led to seven provisional creatinine strata (Table 1). BUN and BUN/ALB showed the largest distributional differences at each adjacent boundary: unadjusted Cliff’s delta ranged from 0.330 to 0.585 for BUN and from 0.229 to 0.614 for BUN/ALB (Table 2). Both retained differences at all six boundaries after adjustment and BH correction across 36 tests. Other markers varied in direction and magnitude; at the boundary between strata 2 and 3, none of the other four markers retained a difference after age adjustment and correction. These comparisons describe the proposed subdivisions but do not establish biological discontinuities or an optimal number of strata.

**Table 2:** Effect sizes for blood-panel differences at adjacent creatinine-stratum boundaries.

Boundary	BUN	PHOS	HCT	BUN/ALB	SAA	NEU/LYM
1|2	0.330 (0.296, 0.367)	−0.151 (−0.189, −0.113)	0.109 (0.070, 0.153)	0.229 (0.189, 0.270)	−0.070 (−0.141, 0.006)	−0.006 (−0.053, 0.040)
2|3	0.462 (0.382, 0.533)	−0.016 (−0.106, 0.077)	−0.152 (−0.242, −0.064)	0.408 (0.321, 0.498)	0.171 (0.014, 0.331)	0.244 (0.138, 0.340)
3|4	0.585 (0.487, 0.677)	0.302 (0.174, 0.428)	−0.418 (−0.530, −0.293)	0.614 (0.525, 0.706)	0.143 (−0.134, 0.413)	0.180 (−0.015, 0.385)
4|5	0.549 (0.398, 0.677)	0.472 (0.321, 0.613)	−0.246 (−0.417, −0.058)	0.589 (0.435, 0.726)	0.174 (−0.193, 0.544)	0.246 (−0.031, 0.521)
5|6	0.511 (0.312, 0.682)	0.298 (0.069, 0.533)	0.090 (−0.126, 0.299)	0.426 (0.209, 0.649)	−0.038 (−0.500, 0.423)	−0.016 (−0.315, 0.282)
6|7	0.559 (0.379, 0.732)	0.473 (0.263, 0.652)	−0.171 (−0.395, 0.041)	0.525 (0.285, 0.729)	−0.140 (−0.551, 0.370)	0.343 (0.085, 0.612)

Values are unadjusted Cliff’s delta (cat-cluster bootstrap 95% CI). Positive values indicate higher measurements in the higher stratum. These descriptive intervals are not multiplicity-adjusted. Separate age-adjusted rank-model effects and BH q-values across 36 boundary-marker tests, together with marker-specific sample sizes, are provided in Supplementary Table S4F; methods are described in Supplementary Methods 1.4, 1.7.

### 3.2 Cohort characteristics and marker trajectories

Among 5,743 episode-anchored visits, 4,698 were assignable to creatinine strata. The remaining 1,045 comprised 1,010 visits without a recorded CRE value and 35 with a suspect CRE value excluded during cleaning. BUN, BUN/ALB, and NEU/LYM generally increased across strata, while HCT was lower in the higher strata. PHOS/CRE and SDMA/CRE declined (Table 3; Figure 2). SAA showed a non-monotonic pattern. Median age increased from 5.9 years in stratum 1 to 14.1 years in stratum 4, with greater variation in the smaller higher strata. Stratum 7 contained 92 visits, with a creatinine median of 10.9 mg/dL (IQR 9.0–12.2). The longitudinal subset comprised 143 episodes from 136 cats. These episode baselines were older than the 4,091 excluded baselines (median 8.8 vs. 5.7 years; *p* < 0.001), indicating selection through clinical follow-up.

**Table 3:** Stratum-wise median and interquartile range of core and derived markers.

Marker	S1	S2	S3	S4	S5	S6	S7
CRE (mg/dL)	1.3 (1.1–1.5)	1.9 (1.8–2.1)	2.5 (2.4–2.7)	3.2 (3.1–3.3)	4.0 (3.8–4.5)	6.1 (5.5–6.6)	10.9 (9.0–12.2)
BUN (mg/dL)	21.0 (18.0–24.0)	23.0 (21.0–26.0)	28.0 (24.0–36.0)	42.0 (34.0–50.8)	59.5 (49.0–72.8)	82.5 (64.0–104.8)	115.0 (93.5–130.0)
PHOS (mg/dL)	4.3 (3.7–4.9)	4.1 (3.6–4.5)	4.0 (3.6–4.7)	4.6 (4.0–5.5)	6.1 (4.6–7.4)	7.7 (5.6–10.5)	10.9 (8.2–13.9)
BUN/ALB (mg/g)	6.5 (5.6–7.5)	7.0 (6.2–8.1)	8.7 (7.1–11.0)	13.1 (11.1–15.6)	20.0 (14.7–23.9)	26.2 (20.2–30.8)	34.3 (27.4–40.7)
HCT (%)	43.0 (38.4–47.3)	44.2 (40.1–48.5)	43.0 (37.0–47.1)	35.8 (31.0–41.4)	31.0 (25.7–38.0)	32.0 (26.1–42.8)	29.1 (25.0–38.0)
PHOS/CRE (mg/mg)	3.21 (2.69–4.00)	2.14 (1.85–2.47)	1.58 (1.40–1.83)	1.39 (1.17–1.72)	1.51 (1.16–1.78)	1.26 (0.98–1.61)	1.05 (0.74–1.27)
SDMA/CRE (μg/mg)	7.86 (6.36–9.38)	5.88 (4.71–7.06)	5.00 (3.85–6.25)	5.14 (3.91–6.09)	5.22 (4.47–6.32)	3.94 (3.49–4.86)	4.22 (2.95–5.17)
NEU/LYM	1.52 (0.95–2.62)	1.51 (0.94–2.58)	2.34 (1.24–3.69)	3.01 (1.76–4.79)	3.98 (2.13–7.26)	3.48 (2.81–7.63)	6.54 (3.50–11.08)
Age (years)	5.9 (2.8–9.3)	6.3 (3.7–9.8)	10.3 (6.1–14.6)	14.1 (9.5–16.8)	10.3 (7.8–14.4)	11.0 (7.8–15.4)	10.2 (7.6–14.4)
SAA (mg/L)	7.8 (5.0–14.0)	7.2 (5.0–11.0)	9.6 (5.6–13.8)	13.0 (6.0–35.8)	24.0 (9.1–39.0)	20.0 (9.1–37.8)	14.5 (5.8–36.5)
Hypokalemia	5.6% (57/1013)	5.6% (36/642)	11.0% (16/145)	15.0% (6/40)	18.4% (9/49)	19.2% (5/26)	27.1% (16/59)

Values are median (IQR) unless otherwise indicated. Hypokalemia denotes K < 3.5 mmol/L and is reported as a percentage of visits with measured K. Sample sizes for each marker and stratum are provided in Supplementary Table S4C.

**Figure 2:**
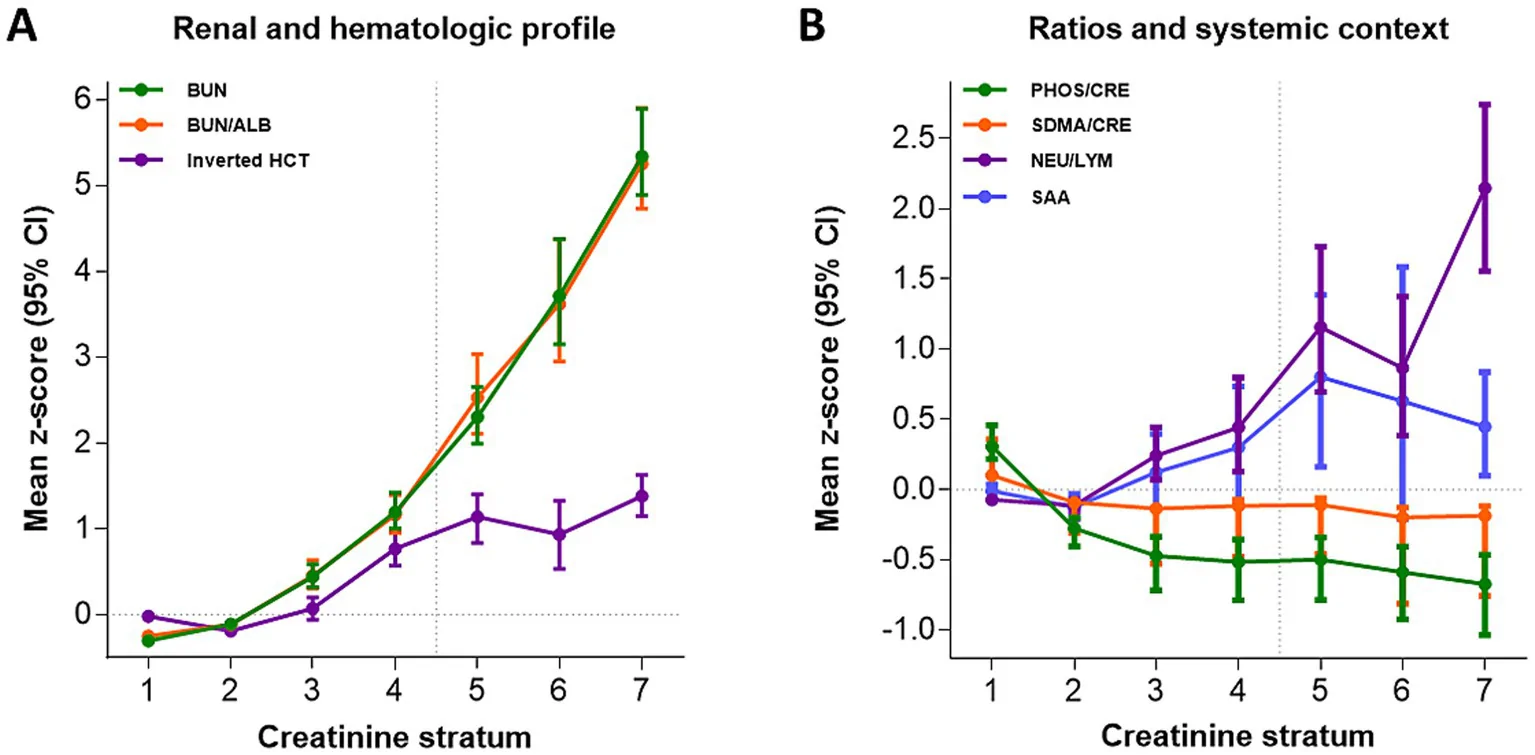
Blood-panel profiles across creatinine strata. **(A)** BUN, BUN/ALB, and inverted HCT. **(B)** PHOS/CRE, SDMA/CRE, NEU/LYM, and SAA. Points show stratum means of z-scores standardized across the cleaned, creatinine-stageable sample; error bars show cat-cluster bootstrap 95% confidence intervals. HCT z-scores were multiplied by −1, so higher values indicated lower HCT. Lines connect stratum means. The vertical dotted line separates strata 4 and 5 (>3.5 mg/dL CRE). Sample sizes are provided in Supplementary Table S4C. Available SAA measurements ranged from 8 to 892 visits per stratum, with only 8 in stratum 6; estimates in sparsely sampled strata should be interpreted cautiously. BUN, blood urea nitrogen; ALB, albumin; HCT, hematocrit; PHOS, phosphorus; CRE, creatinine; SDMA, symmetric dimethylarginine; NEU, neutrophils; LYM, lymphocytes; SAA, serum amyloid A.

Future-only slopes used a median of five measurements (IQR 3–8) over 0.83 years (IQR 0.58–1.48). The median slope was −0.026 mg/dL/year (IQR − 0.522 to 0.417), with a median individual regression R^2^ of 0.452 (IQR 0.150–0.799) (Supplementary Table S4J; Supplementary Code).

### 3.3 Candidate screening and baseline-inclusive associations

The 33-marker screen identified different patterns across cross-sectional association, creatinine slope, and within-episode tracking (Figure 3). PHOS/CRE had a strong inverse cross-sectional association with creatinine (ρ = −0.850; *n* = 4,667). In the baseline-inclusive longitudinal screen, BUN/CRE showed the strongest slope association (ρ = 0.332; *n* = 132; 95% CI 0.151–0.499; q = 0.005). PHOS/CRE (ρ = 0.276; *n* = 131; 95% CI 0.094–0.451) and SDMA/CRE (ρ = 0.324; *n* = 110; 95% CI 0.137–0.531) also met correction within the 33-marker family (both q = 0.025). These three leading markers all used creatinine as their denominator, prompting closer examination of their relationship with the slope calculation.

**Figure 3:**
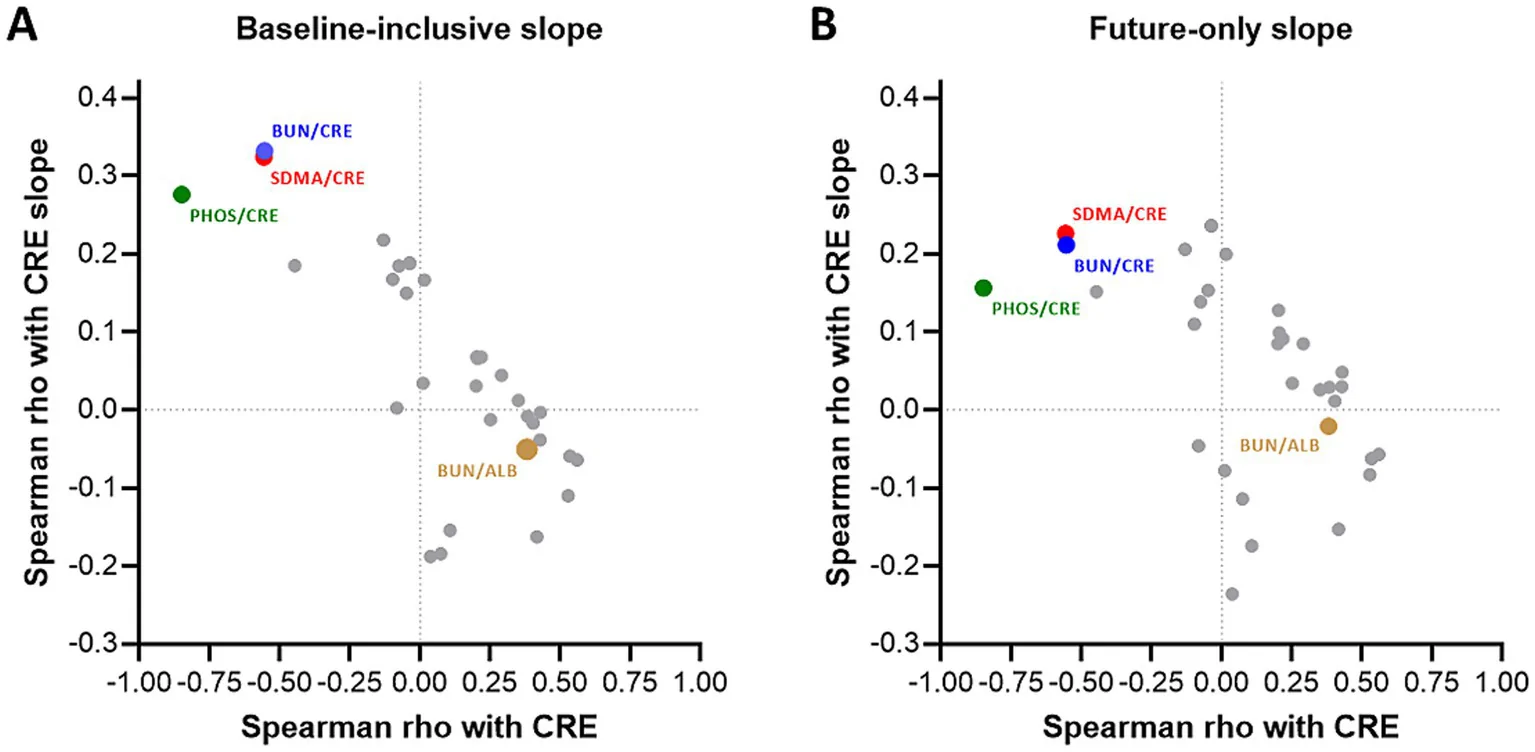
Exploratory screen of 33 blood-panel markers. Each point represents one marker. The x-axis shows its signed Spearman correlation with CRE, and the y-axis shows the correlation of its baseline value with **(A)** the baseline-inclusive CRE slope or **(B)** the future-only CRE slope. BUN/CRE, PHOS/CRE, SDMA/CRE, and BUN/ALB are labeled and colored; other markers are grey. Colors identify selected markers, not statistical significance. Screening statistics and sample sizes are provided in Supplementary Table S1; the comparison of correlation attenuation is provided in Supplementary Table S2. BUN, blood urea nitrogen; CRE, creatinine; PHOS, phosphorus; SDMA, symmetric dimethylarginine; ALB, albumin.

### 3.4 Effects of excluding the baseline visit

BUN/CRE, PHOS/CRE, and SDMA/CRE also had negative median within-episode correlations with creatinine (−0.691, −0.783, and −0.543, respectively). Their baseline measurements therefore had two links to the outcome: creatinine was the denominator of each ratio and was also included in the baseline-inclusive slope. We repeated the analysis after excluding the baseline visit to examine associations with subsequent changes.

Removing baseline reduced absolute correlations by an average of 0.113 for the three creatinine-denominator ratios, compared with −0.007 for the other 27 nonredundant markers (difference 0.120; 95% CI 0.038–0.198). The difference was similar under alternative marker sets and data-cleaning approaches (Supplementary Table S2). Future-only correlations were ρ = 0.212 for BUN/CRE (*n* = 132; q = 0.139), 0.156 for PHOS/CRE (*n* = 131; q = 0.279), and 0.226 for SDMA/CRE (*n* = 110; q = 0.139). None met correction in the future-only 33-marker screen. The greater reduction for these ratios was consistent with shared-baseline coupling, although excluding a visit also changed slope precision.

Baseline CRE itself was negatively correlated with the baseline-inclusive slope (ρ = −0.232; 95% CI -0.416 to −0.042; *n* = 132). Its correlation with future-only slope was weaker (ρ = −0.089; 95% CI -0.271 to 0.085) (Supplementary Table S3H).

### 3.5 Ratio associations within early and advanced strata

In strata 1–2, PHOS/CRE was positively associated with the future-only slope (ρ = 0.312; *n* = 91; 95% CI 0.073–0.511), as was SDMA/CRE (ρ = 0.319; *n* = 79; 95% CI 0.086–0.506). After adjustment for age and baseline creatinine, both associations remained positive but did not meet the 13-marker correction (partial r = 0.229 and 0.269, respectively; both q = 0.072). BUN/CRE and BUN/ALB showed no clear unadjusted associations in this subset.

For PHOS/CRE, future-only correlations were 0.312 in strata 1–2, 0.201 in strata 3–7, and 0.156 in the pooled cohort. The largest observed correlation was in the lower-stratum subset, although these subgroups were examined *post hoc* and no interaction was formally tested.

### 3.6 Adjusted associations and added value of ratios

PHOS/CRE declined from a median of 3.21 in stratum 1 to 1.05 in stratum 7, and SDMA/CRE also declined (Supplementary Figures S1, S2). Because creatinine is the denominator, these patterns were examined alongside adjusted associations and models containing the component analytes.

In the full longitudinal cohort, age- and baseline-creatinine-adjusted future-only associations did not meet correction for BUN/CRE (r = 0.181; q = 0.180), PHOS/CRE (r = 0.158; q = 0.250), or SDMA/CRE (r = 0.213; q = 0.122). PHOS, Ca × PHOS, and ALB/GLOB had the lowest q-values in this analysis (all q = 0.051), but also did not meet the threshold.

In rank-based component models, adding PHOS to age and CRE was associated with future-only slope (*p* = 0.011; q = 0.023 across the two numerator tests), whereas adding PHOS/CRE to that model was not (*p* = 0.549). The PHOS association was not retained in the original scale model (*p* = 0.215). SDMA/CRE did not demonstrate added value beyond age, CRE, and SDMA in the full-cohort rank model (*p* = 0.065; q = 0.130). Flexible models and cross-validation likewise did not show consistent improvement from either ratio (Supplementary Tables S3D,I; Supplementary Code).

In the smaller strata 3–7 subset, PHOS/CRE added information in the rank-based component model (*n* = 40; *p* = 0.020; q = 0.040 across the two ratio tests), but not in the original-scale model (*p* = 0.257). This model dependence limits a general claim of added value.

BUN/ALB increased from a median of 6.5 in stratum 1 to 34.3 in stratum 7. It tracked creatinine within episodes, with a median ρ of 0.500 and positive correlations in 66.7% of evaluable episodes (*n* = 72). Baseline BUN/ALB was not associated with either the baseline-inclusive slope (ρ = −0.051; *p* = 0.628; *n* = 111) or the future-only slope (ρ = −0.021; *p* = 0.834). BUN/ALB mainly tracked concurrent blood-panel values and within-episode change.

### 3.7 Systemic blood-panel patterns

SAA, NEU/LYM, and ALB/GLOB were weakly correlated with CRE, PHOS/CRE, and SDMA/CRE (all |ρ| < 0.2; Figure 4). Correlations among the systemic markers were more apparent: SAA correlated positively with NEU/LYM (ρ = 0.221) and negatively with ALB/GLOB (ρ = −0.267). The weak associations with CRE persisted after age adjustment (partial r = −0.044 for SAA, −0.016 for NEU/LYM, and 0.108 for ALB/GLOB). Pairwise coefficients, sample sizes, and age-adjusted results are provided in Supplementary Tables S4G,K. Unadjusted correlations are also shown in Supplementary Figure S3.

**Figure 4:**
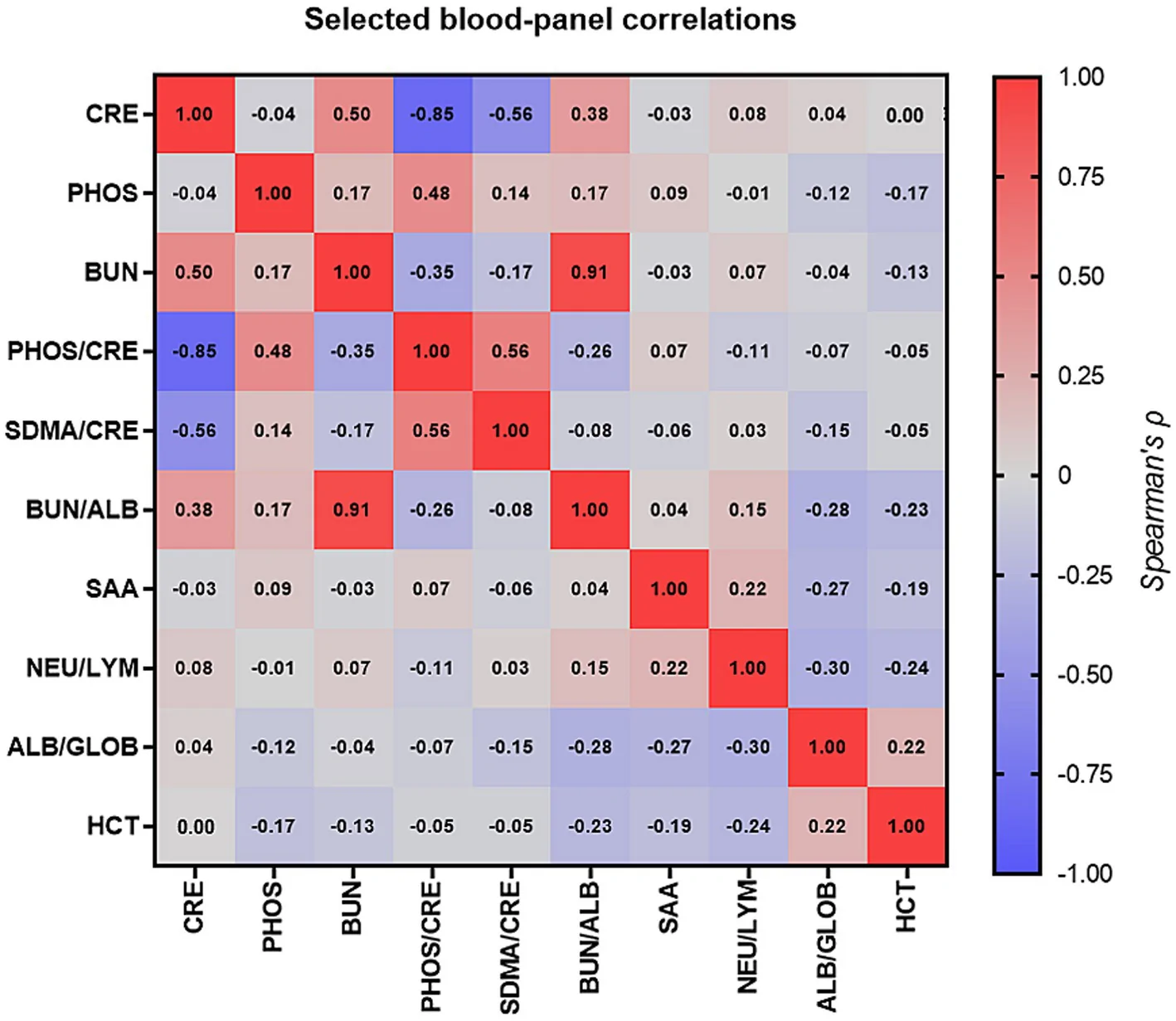
Relationships among selected blood-panel markers. Cells show pairwise-complete Spearman correlation coefficients from the cleaned, episode-anchored visit sample. Red indicates positive correlations and blue indicates negative correlations. Pairwise sample sizes are provided in Supplementary Table S4G. CRE, creatinine; PHOS, phosphorus; BUN, blood urea nitrogen; SDMA, symmetric dimethylarginine; ALB, albumin; SAA, serum amyloid A; NEU, neutrophils; LYM, lymphocytes; GLOB, globulin; HCT, hematocrit.

### 3.8 Potassium and mineral safety flags

Among visits with potassium measurements, the prevalence of hypokalemia increased from 5.6% in stratum 1 to 27.1% in stratum 7, while median potassium changed little (Table 3). These proportions apply to tested visits, because potassium was not measured uniformly across the cohort. PHOS decreased slightly across strata 1–3 before rising in strata 4–7. This non-monotonic pattern, together with the predominance of visits in strata 1–2, helps explain its near-zero pooled correlation with CRE (ρ = −0.041). Ca × PHOS similarly had little overall correlation with CRE (ρ = 0.016), despite higher medians in the higher strata. Electrolyte and mineral findings remained relevant to the clinical questions motivating the study (18–20). Table 4 summarizes the observed associations of the selected markers.

**Table 4:** Summary of marker role classification.

Marker	Baseline-inclusive screen role	Multiplicity	Interpretation
BUN/CRE	Strongest slope association	q = 0.005 Future-only: q = 0.139	Conventional ratio; no clear adjusted future-only association
PHOS/CRE	Positive slope association	q = 0.025 Future-only: q = 0.279	Exploratory early-stratum association; age + CRE adjusted q = 0.072; added value not established
SDMA/CRE	Positive slope association	q = 0.025 Future-only: q = 0.139	Exploratory early-stratum association; age + CRE adjusted q = 0.072; added value not established
BUN/ALB	No slope association	q = 0.910 Future-only: q = 0.860	Current values and within-episode tracking
SAA, NEU/LYM, ALB/GLOB	Not evaluated as a combined slope predictor	Not applicable	Clinical context from systemic markers; added clinical benefit untested
Potassium, Ca × PHOS	Limited value as a single continuous severity summary	Not applicable	Prompts for electrolyte and mineral review

Roles are exploratory. Screening q-values refer separately to the baseline-inclusive and future-only 33-marker families.

## 4 Discussion

### 4.1 Clinical observations and retrospective findings

This study began with a familiar clinical question: what differs between cats whose creatinine values place them in the same broad range? In the clinical experience behind this study, accompanying PHOS, SDMA, inflammatory, and hematologic findings could lead to different interpretations of the current condition and changes during follow-up. We used finer creatinine strata to examine these observations in routine-care data, with IRIS ranges as the clinical reference. The retrospective analysis identified accompanying patterns and tested whether selected ratios also related to subsequent CRE change.

The markers behaved differently. BUN/ALB mainly described current values and within-episode changes, while systemic markers showed patterns weakly related to creatinine. PHOS/CRE and SDMA/CRE had positive associations with subsequent creatinine change in strata 1–2, but their adjusted results did not establish prognostic value. The clearest statistical finding concerned the baseline measurement: excluding it reduced absolute slope correlations more for the three CRE-denominator ratios than for the other markers. Spurious associations involving ratios and mathematical coupling between initial values and subsequent change are established statistical problems (21, 22). Regression to the mean can further complicate interpretation (23). The present results document their practical relevance in a large feline routine-panel dataset, where apparently promising ratio signals depended on how the outcome was calculated.

### 4.2 Interpreting finer strata and selected ratios

The seven strata were intended to describe variation within familiar creatinine ranges. BUN and BUN/ALB showed consistent adjacent-boundary differences, while the other markers varied more. The subdivisions made clinical observations explicit, but the same cohort informed their selection and evaluation. Their clinical value depends on whether these patterns are reproducible and improve assessment beyond broader creatinine ranges.

Ratios were included because clinicians often interpret measurements in relation to one another. PHOS/CRE asks how PHOS compares with the accompanying CRE value, but its strong inverse association with CRE makes the denominator central to its interpretation. Mineral handling, hydration, lean mass, diet, and treatment may also affect these measurements (24–26). PHOS showed a model-dependent association with subsequent CRE slope, present in the rank-based model but not in the original-scale model. PHOS/CRE did not consistently improve on the component model. The descriptive convenience of a ratio should be distinguished from evidence that it predicts subsequent change.

SDMA/CRE similarly describes the relative levels of two filtration-related measurements. A higher ratio indicates more SDMA relative to CRE, which may direct attention to discordant results when reviewing a blood panel. Discordance between these markers has been reported in other settings (27, 28), and differences in muscle mass or hydration may contribute. The positive association in strata 1–2 observed here did not meet the multiple-testing correction threshold after adjustment for age and baseline creatinine, and full-cohort component-model comparisons did not demonstrate consistent additional value. The reason for discordance therefore remains a clinical question rather than a diagnosis supplied by the ratio.

BUN/ALB behaved differently. It increased across strata and tracked creatinine within episodes, but its baseline value showed no clear association with subsequent slope. Azotemia, protein catabolism, hydration, and nutritional or acute-phase status may contribute to BUN/ALB, although these mechanisms were not measured here. Reading them together may help describe the current pattern, while the component values remain necessary to understand why the ratio changes. BUN/CRE also requires this contextual interpretation because hydration and prerenal influences can affect its value. A marker may describe the current blood panel without predicting the subsequent trajectory.

### 4.3 Systemic markers and safety flags in individual assessment

Systemic markers were included because the clinical assessment of a cat with kidney-related findings extends beyond filtration. A stable CRE value does not answer whether accompanying inflammation, altered protein measurements, or hematologic abnormalities have changed. The weak correlations of SAA, NEU/LYM, and ALB/GLOB with CRE were consistent with accompanying systemic findings that were not closely reflected in CRE. These correlations, however, do not establish biological independence or added clinical benefit.

SAA was more strongly correlated with NEU/LYM and ALB/GLOB than with CRE, supporting further examination of these systemic findings together. These measurements are nonspecific and require interpretation alongside the history, examination, and other test results. Inflammation, oxidative stress, and biological aging provide a broader context for studying such patterns (15, 16, 29, 30). Hematologic findings also matter because CKD-associated anemia can require specific management (31). Potassium and Ca × PHOS were retained for a related practical reason: an abnormal electrolyte or mineral result may warrant attention even when it contributes little to a continuous severity trend. The increasing proportion of hypokalemic results illustrated this distinction. Together, these observations support reviewing filtration-related, systemic, and electrolyte findings when assessing an individual cat. Whether this joint interpretation improves clinical decisions remains to be tested.

### 4.4 Limitations and future validation

This single-hospital retrospective study examined blood-panel patterns across creatinine ranges in cats receiving routine care. Clinical diagnoses were unavailable, preventing confirmation of disease status within each stratum and evaluation of the findings specifically in cats with confirmed CKD. Information on treatment, diet, and other clinical factors was also unavailable or incomplete. The longitudinal subset was older and selected through clinical follow-up, limiting generalizability. Accounting for repeated observations within cats does not remove this selection bias.

Measurements were incomplete and clinically selected. Cats with SAA, SDMA, or potassium measurements were older than those without the corresponding measurement, and analysis of available results cannot remove testing-selection bias. The origins of suspect test-field values could not be verified against the source records. Incomplete historical assay documentation also limited assessment of measurement consistency (Supplementary Methods 1.1, 1.3).

Creatinine slope describes a laboratory trajectory rather than confirmed CKD progression. Hydration, lean mass, acute illness, treatment, measurement error, and regression to the mean may affect it, and a single linear slope can miss nonlinear changes. Excluding the baseline visit removes the shared measurements, but does not eliminate dependence on the starting CRE level or regression to the mean. The weak full-cohort correlation between baseline CRE and future-only slope does not rule out these effects. Exclusion also reduces the number of measurements and may affect slope precision. Future studies should relate the observed patterns to clinical outcomes.

The marker screen and subsequent analyses were exploratory, and the clinically proposed boundaries were refined and evaluated in the same cohort. The smaller higher strata and longitudinal subsets limited precision. External validation should use standardized sampling and record body and muscle condition, urinalysis, blood pressure, treatment, and outcomes. The next clinical question is whether interpreting these findings together improves assessment of an individual cat beyond existing clinical information.

## 5 Conclusion

Clinical observations guided a retrospective examination of accompanying blood-panel findings across creatinine ranges. Systemic and electrolyte findings did not consistently track CRE, supporting their consideration alongside filtration-related measurements when assessing an individual cat. However, associations with the current blood panel did not necessarily extend to subsequent CRE change, and apparent ratio signals were sensitive to inclusion of the baseline measurement. Whether interpreting these findings together improves clinical assessment requires prospective validation.

## Data Availability

The datasets presented in this article are not readily available because the underlying raw clinical records are subject to institutional, owner-privacy, and data-use restrictions. Requests for access should be directed to the corresponding author and will require appropriate institutional approval and permission from the data-providing institution. Summary datasets are included in the article and its Supplementary material. The analysis code, marker formulae, environment information, and validation records are provided in the accompanying Supplementary Code package; no patient-level records are included in that package. Requests to access the datasets should be directed to HS, hc.shin@greatmomo.com.
