# Supplementary material for: Blood-panel patterns across feline creatinine strata and shared baseline effects on ratio associations
Source: Front Vet Sci. 2026 Oct 22;13:1972280. doi: 10.3389/fvets.2026.1972280 (PMC13736241; doi:10.3389/fvets.2026.1972280)
Supplement: Supplementary file 1 [file Data_Sheet_1.DOCX]

Supplementary Material

# 1 Supplementary Methods

## 1.1 Data cleaning

Visits required valid dates and patient identifiers. Based on dataset-specific recording patterns, CRE values of 50, 100, 200, or 300 mg/dL and ALB values of 1, 5, or 10 g/dL were flagged as suspected test-field errors and set to missing. These rules affected 93 CRE entries and 185 ALB entries across 191 of 7,094 visits (2.69%). Nonpositive HCT/HGB values were also set to missing; other measurements and visit positions were retained. Derived markers were recalculated. Alternative cleaning excluded whole episodes with suspect CRE, excluded CRE/ALB on visits with urine specific gravity, or retained ALB candidates without supporting evidence. Supporting evidence was urine specific gravity, suspect CRE, or disagreement with the rounding interval implied by recorded ALB/GLOB × GLOB; 176 ALB candidates had such support and nine did not (Supplementary Table S4). Reconstruction sensitivity analyses used CRE = BUN/(recorded BUN/CRE) and ALB = (recorded ALB/GLOB) × GLOB without rounding. SAA was analyzed numerically; 463 of 1,953 recorded values (23.7%) were exactly 5 mg/L, and reporting resolution around 4-5 mg/L was uncertain. BUN was retained as recorded: 38 of 5,614 values were exactly 130 mg/dL and six exceeded 130 mg/dL, leaving possible upper-end reporting limits unresolved.

## 1.2 Episode sampling and creatinine slopes

Episodes were separated by gaps >180 days. Cross-sectional analyses used the first three visits per episode; a sensitivity analysis used the first stageable visit per cat. Within-cat clustering addressed pseudoreplication. Longitudinal episodes required ≥4 valid CRE measurements over ≥0.5 years, with markers taken from the first visit without later-value substitution. Ordinary least squares slopes (mg/dL/year) used all episode measurements, without the three-visit cap. Future-only slopes excluded baseline and required ≥3 subsequent measurements on ≥2 dates. Sensitivity analyses used the first qualifying episode per cat, Theil-Sen and first-to-last slopes, and longer follow-up. Leading ratios were also assessed within strata 1-2 and 3-7. Measurement counts, follow-up intervals, slope distributions, and individual regression R^2^ are summarized in Supplementary Table S4. Follow-up intervals span the measurements used in each slope fit. Baseline CRE-slope correlations were examined separately, with and without age adjustment (Supplementary Table S3).

## 1.3 Sampling and measurement availability

Analyses used available measurements without imputation. Testing groups were compared at the first visit per cat, and longitudinal inclusion groups at episode baseline (Supplementary Table S4). Hypokalemia percentages used measured K values as the denominator; marker-specific counts are in Supplementary Table S4.

## 1.4 Stratum comparisons and figure summaries

Six adjacent boundaries were assessed using BUN, PHOS, HCT, BUN/ALB, SAA, and NEU/LYM. Table 2 reports unadjusted Cliff’s delta with 95% CIs from 999 cat-cluster bootstrap resamples; positive values indicate higher measurements in the higher stratum. Age-adjusted boundary models are described in Section 1.7. Figure 2 shows stratum means of z-scores standardized using each marker’s available values across all 4,698 cleaned, creatinine-stageable visits (sample SD), with cat- cluster bootstrap 95% CIs; HCT z-scores were multiplied by -1.

## 1.5 Candidate markers

Review of routine analytes, ratios, and products yielded 38 markers with direct or conditional definitions: 33 were directly calculable for screening, and five required proxy or conditional definitions and were analyzed separately (Supplementary Table S1). Proxy inputs included TP = ALB + GLOB and MCHC = 100 × HGB/HCT; historical NEU/LYM unit documentation was incomplete.

## 1.6 Marker screening and shared baseline comparison

Spearman correlations assessed associations with CRE, both slope definitions, and within-episode tracking. Tracking used the median episode correlation, requiring ≥3 paired observations with nonconstant values.

Attenuation, |ρ baseline-inclusive| - |ρ future-only|, was compared between BUN/CRE, PHOS/CRE, and SDMA/CRE and the remaining markers. The 30-marker set excluded (BUN/ALB) × PHOS, GLOB/ALB, and -(ALB/GLOB) from the 33-marker screen. The original reconstructed 27-marker set excluded ALB/GLOB, (BUN/ALB) × PHOS, BUN × [-(ALB/GLOB)], Ca/PHOS, Ca×PHOS, and log1p(SAA)/ALB. This post hoc comparison used 999 joint cat-cluster bootstrap resamples to estimate 95% CIs for the difference in mean attenuation (Supplementary Table S2).

## 1.7 Rank models and multiple testing

Rank regression used cat-cluster-robust standard errors and two-sided t tests. Partial correlations used Pearson correlations between residuals after adjusting ranked markers and outcomes for ranked age and/or baseline CRE, with an intercept, in complete cases. Sensitivity analyses used Spearman residual correlations; bootstrap CIs used 999 cat-cluster resamples.

Benjamini-Hochberg correction covered 33 tests per cross-sectional or slope screen, up to 13 per longitudinal subset and adjustment specification, and 36 boundary comparisons. Models with n <15 were omitted. Boundary models used rank-fraction outcomes, a higher-stratum indicator, and an age spline (3 degrees of freedom), with cat-cluster-robust inference. Independent-group comparisons used Mann-Whitney U tests. Baseline CRE-slope comparisons used a separate family of six comparisons (three subsets × two slopes) for each adjustment specification.

## 1.8 Systemic marker comparisons

Pairwise-complete Spearman correlations used same-visit measurements from the cleaned episode-anchored sample. Age-adjusted partial rank correlations used the stageable sample, with a three-degree-of-freedom age spline as a sensitivity analysis. BH correction covered 15 pairs per adjustment. Results are provided in Supplementary Table S4.

## 1.9 Component models and cross validation

Future-only slope models added PHOS or SDMA to age and CRE, then the corresponding ratio. The main models used rank fractions (rank/[n + 1]) for the outcome and predictors. Sensitivity models used the original-scale slope with ranked predictors, or the original scale for both outcome and predictors. Flexible models added squared and cubic CRE and numerator terms. Coefficient CIs and tests used cat-cluster-robust standard errors. BH correction covered two numerator or two ratio tests separately within each subset and model specification. Five repeats of five-fold cross-validation separated cats and fitted ranks within training folds. CIs resampled cats from paired out-of-fold errors without model refitting. Stratum and continuous-CRE models were compared descriptively (Supplementary Table S3).

## 1.10 Selected sensitivity results

Greater attenuation of CRE-ratio associations persisted across marker sets and reconstruction analyses. Individual longitudinal significance varied across cleaning and slope specifications (Supplementary Tables S2 and S3). Retaining the nine unsupported ALB candidates left the age- and CRE-adjusted longitudinal results for BUN/ALB and ALB/GLOB unchanged.

# 2 Supplementary Figures

**Supplementary Figure S1. PHOS/CRE distribution across creatinine strata.** Boxes show the median and interquartile range; whiskers extend to the most extreme observations within 1.5 times the interquartile range. Outliers are not shown. Sample sizes are provided in Supplementary Table S4. PHOS, phosphorus; CRE, creatinine.

**Supplementary Figure S2. SDMA/CRE distribution across creatinine strata.** Boxes show the median and interquartile range; whiskers extend to the most extreme observations within 1.5 times the interquartile range. Outliers are not shown. Sample sizes are provided in Supplementary Table S4. SDMA, symmetric dimethylarginine; CRE, creatinine.

**Supplementary Figure S3. Correlations among blood-panel markers.** Values are pairwise-complete Spearman correlation coefficients (ρ) from the cleaned first-three-visits-per-episode sample. Red and blue indicate positive and negative correlations, respectively. Pairwise sample sizes are provided in Supplementary Table S4.
